# Virulence characterization of *Klebsiella pneumoniae* and its relation with ESBL and AmpC beta-lactamase associated resistance

**Published:** 2020-04

**Authors:** Elghar Soltani, Alka Hasani, Mohammad Ahangarzadeh Rezaee, Tahereh Pirzadeh, Mahin Ahangar Oskouee, Akbar Hasani, Arezoo Noie Oskouie, Ehsan Binesh

**Affiliations:** 1 Immunology Research Center, Tabriz University of Medical Sciences, Tabriz, Iran; 2 Department of Bacteriology and Virology, School of Medicine, Tabriz University of Medical Sciences, Tabriz, Iran; 3 Drug and Applied Research Center, Tabriz University of Medical Sciences, Tabriz, Iran; 4 Department of Infectious Disease, School of Medicine, Shahroud University of Medical Science, Shahroud, Iran

**Keywords:** *Klebsiella pneumoniae*, Virulence, Antibiotic resistance, Extended spectrum beta-lactamase, AmpC β-lactamase

## Abstract

**Background and Objectives:**

Trend analysis reveals that *Klebsiella pneumoniae* has witnessed a steep enhancement in the antibiotic resistance and virulence over the last few decades. The present investigation aimed at a comprehensive approach investigating antibiotic susceptibility including, extended spectrum beta-lactamase (ESBL) and AmpC β-lactamase (AmpC) resistance and the prevalence of virulence genes among the *K. pneumoniae* isolates.

**Materials and Methods:**

Sixty-one *K. pneumoniae* isolates were obtained from various clinical infections. Antimicrobial susceptibility was performed by disk diffusion method. The Mast® D68C test detected the presence of ESBLs and AmpCs phenotypically, and later presence of ESBL and AmpC genes was observed by polymerase chain reaction (PCR). Multiplex-PCR was performed to investigate various virulence genes.

**Results:**

Amongst 61 *K. pneumoniae* isolates, 59% were observed as ESBL and 14.7% as AmpC producers. All ESBL producers were positive for *bla*_ CTX-M-15_ , while *bla*_ CTX-M-14_ was observed in 54.1% isolates. The frequency of AmpC genes was as follows: *bla*_ CMY-2_ (60.7%) and *bla*_ DHA-1_ (34.4%). The most frequent virulence genes were those encoding enterobactin and lipopolysaccharide. Presence of *mrkD* was associated with *bla*_ DHA-1_ gene, while *bla*_ CMY-2_ significantly (p≤0.05) correlated with the presence of *iutA* and *rmpA* virulence genes. *bla*_ DHA-1_ positive isolates had urine as a significant source, while *bla*_ CMY-2_ positive isolates were mainly collected from wound exudates (p≤0.05).

**Conclusion:**

Our results highlight that ESBL and AmpC production along with a plethora of virulence trait on *K. pneumoniae* should be adequately considered to assess its pathogenesis and antibiotic resistance.

## INTRODUCTION

The vast credentials to acquire multiple antibiotic resistance mechanisms and express virulence traits scrupulously have led *Klebsiella pneumoniae* to attain medical importance amongst other opportunistic pathogens (1). Amidst antibiotic resistance mechanisms, high prevalence of extended-spectrum β-lactamases (ESBLs) and plasmid-mediated AmpC β-lactamases (AmpCs) is the widely known concern (2). AmpC β-lactamases are clinically important cephalosporinases encoded on the chromosomes and plasmids of many of the Enterobactericeae bacteria, especially in *K. pneumoniae.* Like the chromosomally determined AmpC β-lactamases, the plasmid-mediated enzymes confer resistance to a broad spectrum of β-lactams including penicillins, oxyimino--cephalosporins, cephamycins, and aztreonam (variably) (2). ESBLs are the derivatives of common β-lactamases that have undergone one or more amino acid substitutions near the active site of the enzyme, thus increasing their affinity and the hydrolytic activity against third-generation cephalosporins and monobactams. Extensive use of newer cephalosporins and the emerging resistance towards them has been the strong factor for the evolution of newer β-lactamases. The ESBL enzymes can be classified on the basis of their primary structure into four molecular classes (A through D), or on the basis of their substrate spectrum and responses to inhibitors into a larger number of functional group (3). AmpCs named with inconsistency typical of β-lactamase nomenclature according to the resistance produced to cephamycins (CMY), cefoxitin (FOX) and moxalactam (MOX) or latamoxef (LAT). Type of β-lactamase, such as AmpC type (ACT) or Ambler class C (ACC), and to the site of discovery, such as the Miriam Hospital in Providence, R.I. (MIR-1) or Dhahran hospital in Saudi Arabia (DHA) are other form of naming them. These enzymes have been identified throughout the world, with CMY-2 and DHA-1 being the most prevalent (4).

The ability of *K. pneumoniae* to cause various infections is also attributed to the expression of arrays of virulence genes. In fact, published literature suggests that synchronization between antibiotic resistance and virulence traits may lead to the treatment failure in *K. pneumoniae* and *E. coli* related infections (1). Though emergence of resistant bacteria and evolution of pathogenetic mechanism may occur at different times however, it has been postulated that both the processes at the biological point of view are necessary for any bacteria to survive in different environments. Moreover, resistance to antibiotics may have an effect on virulence (5). Virulence factors related to the pathogenicity of *K. pneumoniae* are numerous and have a wide range of activities, spanning from bacterial colonization to virulence, including capsule, fimbriae, lipopolysaccharides (LPS), efflux pumps and siderophores (enterobactin, aerobactin, salmochelin, and yersiniabactin) (6). Different virulence-associated genes including, capsules *(magA, k2A, wcaG)*, those encoding lipopolysaccharides *(wabG, uge, ycfM)*, regulators of hypermucoviscosity *(rmpA, magA)*, adhesins *(fimH, mrkD, kpn)*, iron acquisition systems *(iutA, iroN)*, enterobactin *(entB)*, allantoin metabolism *(allS)* and other virulence genes like *hly* and *cnf-1* allow the bacteria to overcome innate host immunity (6). Earlier studies documented the role of classical *K. pneumoniae* strains in causing serious infections in immunocompromised individuals. However, since the 1980s *K. pneumoniae* hyper-virulent strains that can cause serious infections in otherwise healthy individuals have been recognized. It has been suggested that though the carriage and expression of drug resistance may not enhance the virulence nevertheless, make bacteria more difficult to treat (7). Possession of a capsule not only allow the bacteria to evade the host immune response but also provides antibacterial peptides. The same holds true for LPS. There is a significant heterogeneity in *K. pneumoniae* isolates and several virulence factors less well characterized remain to be discovered to provide more comprehensions about the characterization of this pathogen in various infections and identify different target sites for treating these infections (7).

Diversity in clinical features of *K. pneumoniae* infections is linked with a number of expressed virulence factors, antibiotic resistance and nature of the bacterium (8), thus an increased understanding of the pathogenesis is vital. The distribution of *K. pneumoniae* is related to geographical regions and the type of infections. Moreover, the third generation cephalosporins are therapeutic agents crucial for the treatment of severe infections caused by *K. pneumoniae* therefore, studying of both processes might provide better understanding of the relationship between β-lactam resistance and virulence (1). Thus, we aimed to assess virulence traits of *K. pneumoniae*, and the production of ESBL or AmpC so as to gain an insight into any relation between antibiotic resistance and virulence profile.

## MATERIALS AND METHODS

### Bacterial strains and phenotypic characterization.

In this prospective study, a total of 61 consecutive non-duplicate *K. pneumoniae* isolates were collected from various clinical specimens including, urine, blood, wound, endotracheal aspirate (EA), and other body fluids from patients admitted to the University-based teaching hospital, Sina Hospital in Tabriz, Iran from September 2016 through May 2017. These isolates were initially identified using conventional biochemical tests (9), and later by polymerase chain reaction (PCR) as described elsewhere (10). Ultimately, the isolates were stored in tryptic soy broth (TSB, Merck) containing 20% glycerol and kept at −70°C for further use. This study was approved by Ethical Committee of Tabriz University of Medical Sciences, Tabriz [IR.TBZMEDE.REC.1397.058].

### Antimicrobial susceptibility test.

Disk diffusion method was used for performing antimicrobial susceptibility testing as per the guidelines of the Clinical Laboratory Standard Institute (CLSI) and findings were interpreted accordingly (11). Antibiotic disks were purchased from Mast Diagnostics, UK. *E. coli* ATCC 25922 was used as a quality control strain. The following antibiotic disks were used: ciprofloxacin (5 μg), amikacin (30 μg), gentamicin (10 μg), ceftazidime (30 μg), cefotaxime (30 μg), piperacillin-tazobactam (100/10 μg), nitrofurantoin (300 μg) (used only for urinary isolates), imipenem (5 μg), meropenem (5 μg), co-trimoxazole (1.25/23.75 μg) and levofloxacin (5 μg). The isolates that were resistant to at least one antimicrobial agent in three or more of the categories were considered as multidrug-resistant (MDR) (12).

### D68C ESBL and AmpC detection set.

This combination disk method is used for the identification of both ESBL and AmpC β-lactamase production. The detection set comprised of four disks including, disk A (10 μg cefpodoxime as the screening agent), disk B (10 μg cefpodoxime and clavulanate as the ESBL inhibitor), disk C (10 μg cefpodoxime and cloxacillin as the AmpC inhibitor) and disk D (10 μg cefpodoxime in combination with both clavulanate and cloxacillin). Primarily, a bacterial suspension equivalent to the density of 0.5 McFarland opacity standard was inoculated on Mueller-Hinton agar plate according to the manufacturer’s recommendations (MAST-DISCS™ID, UK) and the plate was incubated at 37 °C for 24 h. The results were interpreted by comparing the zone of inhibition around four disks (13).

### DNA isolation and PCR amplification.

DNA was extracted from *K. pneumoniae* isolates using the commercial DNA extraction kit (Stratec Biomedical systems, Birkenfeld, Germany). In summary, a bacterial suspension matched equivalent to 0.5 McFarland was obtained from an overnight culture and centrifuged. DNA was extracted as per the instructions provided in the kit from the pellet and then resolved in 100 μl Tris-EDTA buffer. For all isolates, PCR amplification was conducted with primers that target *bla*_ CTX-M-14_ (14), *bla*_ CTX-M-15_ (15) and *bla*_ DHA-1_ , and *bla*_ CMY-2_ (16) genes.

Totally, 16 virulence genes including *(magA, k2A, rmpA, wcaG, uge, cnf-1, entB, ycfM, kpn, wabG, fimH, mrkD, iutA, iroN, hly* and *allS)* were identified by performing four separate multiplex PCR, and using primers as described previously (6). PCR conditions for four multiplex PCR consisted of an initial denaturation at 95 ºC for 5 minutes, 30 cycles of 1 min at 94 °C, 1 min at 58 °C and 1 min 72 °C, followed by final extension step for 10 min at 72 °C. The amplified products were electrophoresed on 1% agarose gel and, stained with Cyber safe stain.

### Statistical Analysis.

Variables were compared by χ2 and Fisher’s exact tests using SPSS 20.0 statistical software (IBM Corporation). *P*-value <0.05 were considered as statistically significant.

## RESULTS

During the period of study, 61 *K. pneumoniae* isolates collected from various types of specimens were initially identified by traditional biochemical tests. All isolates had a positivity for the internal transcribed spacer region *(K. pneumoniae 16S-23S)* and were confirmed at the molecular level as *K. pneumoniae*. These isolates were collected from 33 (54.1%) females and 28 (45.9%) males, aged 3 to 89 years, with mean ± SD = 56.7 ± 23.42 years. The majority of the isolates (70.5%) were obtained from hospitalized patients, while the rest (29.5%) from outpatients. The hospital source of these isolates encompassed: Intensive Care Unit (ICU) (36.2%) followed by internal (13.1%), burn (9.8%), urology (9.8%), infectious (6.6%) and emergency (3.3%) wards.

### Prevalence of virulence genes.

Siderophore gene *entB* (enterobactin) was the most prevalent [58 (95.1%)] gene among 16 determined virulence genes. Lipopolysaccharide associated genes *(uge, ycfM and wabG)* were observed in 57 (93.4%), 56 (91.8%) and 54 (88.5%) *K. pneumoniae* isolates, respectively. Adhesin encoding genes such as *kpn* was present in 28 (45.9%) isolates, followed by *mrkD* [17 (27.9%)], and fimH [11 (18%)] genes, encoding for type 3 and type 1 fimbrial adhesin. Other genes such as *iutA* and *iroN* related to iron acquisition systems were detected in 20 (32.8%) and 4 (6.6%) isolates. Among the capsule encoding genes, only *wcaG* was observed in 17 (27.9%) isolates, while no isolate was found positive for *magA* and *k2A* genes. In total, 21.3% *K. pneumoniae* isolates were positive for *rmpA* (hypermucoviscosity) while, following virulence genes had lower prevalence: *cnf-1* (n=3; 4.9%), *allS* (n=2; 3.3%), and *hly* (n=1; 1.6%) (Table 1).

**Table 1.** Distribution of ESBL and AmpC and prevalence of virulence genes in *K. pneumoniae* isolates

**Virulence groups**	**Virulence genes**	**ESBL genes**		**AmpC genes**		**Phenotypic detection**	
		
		***bla*_CTX-M-14_ (n=33)**	***bla*_CTX-M-15_ (n=61)**	***bla*_CMY-2_ (n= 37)**	***bla*_DHA-1_ (n=21)**	**ESBL production (n=36)**	**AmpC production (n= 9)**
Lipopolysaccharides	*uge* (n= 57)	32	57	34	19	34	8
	*ycfM* (n= 56)	30	56	34	20	34	9
	*wabG* (n= 54)	29	54	33	19	33	7
Adhesins	*kpn* (n= 28)	15	28	4	8	20 (*p*=0.05 )	7 (*p*=0.04 )
	*mrkD* (n= 17)	3	17	12	14 (*p*=0.01 )	12 (*p*=0.04 )	0
	*fimH* (n= 11)	9 (*p*=0.04 )	11	7	5	7	0
Iron acquisition systems	*iutA* (n= 20)	14	20	16 (*p*=0.02 )	6	14	1
	*iroN* (n= 4)	1	4	4	1	2	0
Enterobactin	*entB* (n= 58)	31	58	37	20	34	7 (*p*=0.05 )
Allantoin metabolism	*allS* (n= 2)	1	2	1	1	1	0
Capsule encoding genes	*wcaG* (n= 17)	8	17	11	5	8	1
	*magA* (n=0)	0	0	0	0	0	0
	*K2A* (n=0)	0	0	0	0	0	0
Hypermucoviscosity	*rmpA* (n=13)	10	13	11 (*p*=0.04 )	5	12 (*p*=0.005 )	0
Toxins	*cnf-1* (n=3)	1	3	1	1	1	1
	*hly* (n= 1)	1	1	1	0	0	0

### Detection of ESBL- and AmpC-production by the Mast® D68C test.

The prevalence of ESBL and AmpC individually, both ESBL and AmpC- simultaneously and neither ESBL nor AmpC- producing *K. pneumoniae* was as follows: 31 (50.8%), 4 (6.5%), 5 (8.1%) and 21 (34.4%) respectively. Overall, the frequency of ESBL- and AmpC-producing isolates accounted for 59% (n=36) and 14.7% (n=9) respectively.

### Distribution of ESBL and AmpC genes.

All 61 *K. pneumoniae* isolates were characterized for the presence of ESBL and AmpC genes. PCR showed that 100% (n=61) and 54.1% (n=33) isolates were positive for *bla*_ CTX-M-15_ and *bla*_ CTX-M-14_ genes, respectively. Forty-four of 61 (72.1%) clinical isolates with ESBL genes co-harbored AmpC associated genes. Thirty-seven (60.7%) contained *bla*_ CMY-2_ gene while, 21 (34.4%) had *bla*_ DHA-1_ gene. Coexistence of both *bla*_ CMY-2_ , and *bla*_ DHA-1_ genes was detected in 14 (22.9%) isolates. Overall, 17 (27.8%) isolates were not positive for any AmpC genes.

### Correlation of ESBL and AmpC at phenotypic and genotypic levels with virulence characteristics.

Amongst AmpC genes, *bla*_ CMY-2_ was associated with *iutA* and *rmpA* virulence genes, whilst *mrkD* was highly associated with *bla*_ DHA-1_ gene (*p*<0.05). *fimH* was the only virulence gene observed to be associated with *bla*_ CTX-M-14_ ESBL gene. ESBL-producer isolates showed significant correlation with adhesins such as *mrkD* and *fimH* and hypermucoviscosity *rmpA* gene (*p*<0.05). On the other hand, *kpn* as an adhesin and enterobactin virulence genes were predominant among AmpC-producer isolates (*p*<0.05)(Table 1).

When the distribution of ESBL and AmpC genotypes was compared with the phenotypic frequency, among 36 ESBL producers 18 (50%) isolates possessed *bla*_ CTX-M-14_ while, all isolates were positive for *bla*_ CTX-M-15_ . Between 9 *K. pneumoniae* AmpC producers, *bla*_ CMY-2_ was the most frequent genotype (n=5; 55.5%), followed by *bla*_ DHA-1_ (n=3; 33.3%) isolates. Only one (11.1%) isolate co-harbored *CMY-2* and *DHA-1* genes.

### Virulence profiles.

Based on the various combinations of virulence profiles, *K. pneumoniae* isolates could be grouped into twelve profiles (Table 2). Each profile contained 4 to 8 virulence factors. The most predominant profile was profile XII with nine isolates, followed by profile XI. Both predominant profiles included lipopolysaccharide associated genes *(wabG, ycfM* and *uge)*, and enterobactin. The marked feature observed in our investigation was the correlation of AmpC production with profile XI (6 of 9 (66.6%), *p*≤0.05).

**Table 2.** Virulence genes profile of *K. pneumoniae*

	**Virulence profiles**	**No. of virulence genes**	**Frequency of pattern**	**ESBL genes**		**AmpC genes**		**Phenotypic detection**	

				***bla*_CTX-M-14_ (n=33)**	***bla*_CTX-M-15_ (n=61)**	**CMY-2 (n=37)**	**DHA-1 (n=21)**	**ESBL production (n=36)**	**AmpC production (n=9)**
I	*wabG, ycfM, entB, uge, kpn, iutA, rmpA, fimH*	8	2	2	2	2	2	2	0
II	*wabG, ycfM, entB, uge, mrkD, wcaG, rmpA*	7	2	2	2	2	2	2	0
III	*wabG, ycfM, entB, uge, kpn, iutA, wcaG*	7	2	1	2	1	1	1	1
IV	*wabG, ycfM, entB, uge, kpn, iutA, mrkD*	7	2	0	2	1	0	2	0
V	*wabG, ycfM, entB, uge, kpn, iutA, rmpA*	7	3	2	3	3	0	2	0
VI	*wabG, ycfM, entB, uge, kpn, mrkD*	6	2	0	2	1	1	2	0
VII	*wabG, ycfM, entB, uge, kpn, iutA*	6	2	2	2	1	0	0	0
VIII	*wabG, ycfM, entB, uge, mrkD*	5	2	0	2	0	1	1	0
IX	*wabG, ycfM, entB, uge, wcaG*	5	3	2	3	1	1	0	0
X	*wabG, ycfM, entB, uge, iutA*	5	2	2	2	2	2	1	0
XI	*wabG, ycfM, entB, uge, kpn*	5	6	4	6	1	1	6	6*
XII	*wabG, ycfM, entB, uge*	4	9	3	9	7	3	4	0

* Significant association (*p*≤0.05)

### Relation of clinical specimens with virulence genes and ESBL and AmpC beta-lactamase genes.

Clinical source of *K. pneumoniae* was as follows: urine (n=31; 50.8%), wound exudates (n=15; 24.6%), blood (n=8; 13.1%), body fluids including CSF (n=4; 6.6%), and endotracheal aspirates (n=3; 4.9%). Table 3 shows the distribution of virulence factors, β-lactamase production and associated genes in various clinical specimens. Among different virulence genes, *wcaG* was significantly associated with isolates obtained from urine specimens, while *rmpA* and *iutA* genes had an association with isolates collected from wound exudates (*p*≤0.05). *bla*_ CMY-2_ , and *bla*_ DHA-1_ genes were associated with isolates obtained from wound and urine specimens, respectively (*p*≤0.05).

**Table 3.** Prevalence of virulence factors and β-lactamase genes among various clinical specimens

		**No. (%) of clinical specimens**				

		**Urine (n=31)**	**Wound (n=15)**	**Blood (n=8)**	**Body fluid (n=4)**	**EA^a^ (n=3)**
**Virulence factors**	*entB*	31 (100)	13 (86.6)	8 (100)	3 (75)	3 (100)
	*uge*	29 (93.5)	15 (100)	7 (87.5)	3 (75)	3 (100)
	*ycfM*	28 (90.3)	14 (93.3)	7 (87.5)	4 (100)	3 (100)
	*wabG*	28 (90.3)	13 (86.6)	7 (87.5)	3 (75)	3 (100)
	*kpn*	12 (38.7)	8 (53.3)	6 (75)	1 (25)	1 (33.3)
	*iutA*	4 (12.9)	10 (66.6) (*p*=0.02 )	5(62.5)	1 (25)	0
	*mrkD*	8 (25.8)	4 (26.6)	3 (37.5)	1 (25)	1 (33.3)
	*wcaG*	12 (38.7) (*p*=0.04 )	2 (13.3)	1 (12.5)	1 (25)	1 (33.3)
	*rmpA*	3 (9.6)	7 (46.6) (*p*= 0.03 )	3 (37.5)	0	0
	*fimH*	7 (22.5)	4 (26.6)	0	0	0
	*iroN*	3 (9.6)	0	0	1 (25)	0
	*cnf-1*	2 (6.4)	0	0	1 (25)	0
	*allS*	2 (6.4)	0	0	0	0
	*hly*	1 (3.2)	0	0	0	0
ESBL genes	*bla*_CTX-M-14_	15 (48.8)	10 (66.6)	4 (50)	2 (50)	2 (66.6)
	*bla*_CTX-M-15_	31 (100)	15 (100)	8 (100)	4 (100)	3 (100)
AmpC genes	*CMY-2*	14 (45.1)	13 (86.6) (*p*=0.04 )	6 (75)	2 (50)	3 (100)
	*DHA-1*	12 (38.7) (*p*=0.03 )	5 (33.3)	1 (12.5)	1 (25)	2 (66.6)
ESBL production		16 (51.6)	9 (60)	6 (75)	3 (75)	2 (66.6)
AmpC production		5 (16.1)	2 (13.3)	1 (12.5)	1 (25)	0

a EA= Endo-tracheal aspirate

### Antimicrobial susceptibility and virulence factors.

Majority of *K. pneumoniae* isolates (78.7% and 75.4%) showed resistance towards cefotaxime and ceftazidime respectively. More than 60% isolates had resistance towards nitrofurantoin (68.9%), ciprofloxacin (68.9%), co-trimoxazole (67.2%). Resistance of *K. pneumoniae* isolates towards other antibiotics was as follows: piperacillin-tazobactam (57.4%), gentamicin (45.9%), amikacin (39.3%) and levofloxacin (24.6%). Around 24% isolates showed resistance to imipenem and meropenem. Forty-seven (77%) *K. pneumoniae* isolates were recorded as MDR. When antibiotic resistance in *K. pneumoniae* isolates was compared with 16 virulence genes, *iutA* positive isolates showed significant (*p*<0.05) resistance to cefotaxime, ceftazidime, ciprofloxacin, piperacillin-tazobactam, gentamicin, and amikacin. Cefotaxime, ceftazidime, ciprofloxacin, co-trimoxazole, and piperacillin-tazobactam resistance was a significant feature observed in *rmpA* positive isolates (*p*<0.05). Ciprofloxacin and co-trimoxazole resistant strains were significantly associated with *ycfM* positivity followed by cefotaxime and gentamicin resistance associated with *kpn* presence and *mrkD*, which was associated with ciprofloxacin resistance (*p*<0.05). *wcaG* had a high prevalence in MDR isolates (*p*=0.05).

### Antimicrobial susceptibility and its relation with ESBL and AmpC producers.

Isolates positive for *bla*_ CMY-2_ were significantly associated with resistance to ciprofloxacin, amikacin and gentamicin, (*p*<0.05). Among ESBL genes, *bla*_ CTX-M-14_ was significantly associated with resistance to nitrofurantoin (*p*<0.05). ESBL-producing isolates had a significant association with resistance against cephalosporins, ciprofloxacin, and co-trimoxazole (*p*<0.05). *bla*_ CMY-2_ positivity and ESBL-production was a significant feature among MDR strains (*p*<0.05).

## DISCUSSION

*Klebsiella pneumoniae* is an opportunistic pathogen afflicting both community and hospital settings. Similar to other bacteria, this organism has acquired resistance to diverse antimicrobial agents and have modified their virulence to adapt to the host defense systems (8). In fact, it is an important host of AmpC β-lactamase and ESBL enzymes. Bacterial resistance towards beta-lactams via ESBL and AmpC production has increased intensely in human pathogens, causing important mortality and morbidity (2, 3). *K. pneumoniae* also harbor several virulence factors that enable the bacteria to colonize and contribute to its pathogenesis. Virulence elements also provide durability to the organism against an effective host defense and develop the infection particularly in immunologically compromised patients (8). The current research attempted to assess the prevalence, distribution of virulence genes and analyses of β-lactamase resistance among *K. pneumoniae* isolates obtained from various clinical specimens.

In the present investigation, *entB* gene was found the predominant (95.1%) virulence marker. The role of enterobactin in *Klebsiella* virulence is still ambiguous; nevertheless, the presence of *entB* stimulates biofilm development and maturation (6). Lipopolysaccharide associated genes *(uge, ycfM* and *wabG)* are commonly found in *K. pneumoniae* and are considered the basis of its pathogenicity with the genes involved in capsule lipoprotein, external membrane protein and capsule production, respectively (6). In this research, *uge, ycfM* and *wabG* had higher prevalence with 93.4%, 91.8% and 88.5% respectively compared to other virulence genes. These high rates are in accordance with previous studies (6, 8). In the current study, siderophores genes *iutA* and *iroN* were positive in 23 (37.7%) isolates and fimbrial adhesins such as *fimH, mrkD* and *kpn* were detected in 40 (65.5%) *K. pneumoniae* isolates. These results corroborate the findings of previous study (6) and show that these virulence factors are essential for *K. pneumoniae* pathogenicity. In our research, frequency of *cnf-1, allS* and *hly* genes was low in all isolates. Similar observation is presented earlier by Yu et al. (17). The absence of *magA* (specific to K1 capsule serotype), and *k2A* (specific to K2 capsule serotype) genes may be due to no liver abscess specimens in this study. Our results are compatible to a previous study (18).

According to our outcomes, ESBL-producers were more prevalent than AmpC by using Mast®D68C-test which utilizes four disks. Similar information has been presented previously (19). The Mast®D68C-test is an effective and easy method to accomplish for the detection of ESBL production and could also be valuable for the detection of plasmid-encoded AmpC enzymes (sensitivity = 100%) (13). According to Nourrisson et al. (2) reports, when the results of D68C test were compared with those of the double disk test and CLSI confirmatory test for the detection of ESBL, the sensitivity was 90.6% for the synergy test, 87.5% for the CLSI method and only 73.1% for D68C while specificity was 90.2% for the synergy test and 100% for CLSI method and D68C. No false-positive result was observed (specificity = 100%). Though Mast®D68C test has low sensitivity but possess high specificity in comparison to the two phenotypic tests mentioned above for ESBL detection.

The plasmids of *K. pneumoniae* possess many β-lactamase genes including those encoding extended-spectrum β-lactamases and AmpC β-lactamases. In this investigation, PCR detected *bla*_ CTX-M-15_ and *bla*_ CTX-M-14_ in 100% and 54.1% ESBL producing *K. pneumoniae* respectively. Recently, CTX-M type positive strains have been increasingly identified from most parts of the world (20). Comparative to other report (21), incidence of *bla*_ CTX-M-14_ and *bla*_ CTX-M-15_ was too high in our hospital isolates. The higher rates of CTX-M among total ESBL enzymes are most probably associated with high mobilization of the encoding genes (15). Based on our findings, plasmid-mediated AmpC genes, *bla*_ CMY-2_ and *bla*_ DHA-1_ were found in 72.1% *K. pneumoniae* isolates. Various studies from China (22), Turkey (23), Egypt (24), Korea (25) have reported the prevalence of AmpC *K. pneumoniae* varying from 4.2% to 26%. It seems that the rate of plasmid-mediated AmpC β-lactamases is increasing among *Klebsiella* worldwide. It is noteworthy to mention the high frequency of plasmid-mediated AmpC genes in our hospital isolates. The incidence of AmpC and ESBL genes in diverse hospitals depends on certain factors including, antibiotic prescription, carriage rate of bacteria among hospital personnel, type of disinfection used particularly in the ICU ward, and use of invasive procedures such as urinary catheterization (26). Earlier Yum et al. indicated that *bla*_ CMY-2_ was the most common type of AmpC β-lactamase followed by *bla*_ DHA-1_ (21). We found *K. pneumoniae* isolates co-harbored *bla*_ CMY-2_ and *bla*_ DHA-1_ genes which is in agreement with the results of an investigation performed in Korea (21). These β-lactamase enzymes (ESBL and AmpC) even contribute resistance to other antibiotics too (27). Our study also showed that though *K. pneumoniae* possessed both β-lactamases but the majority of resistant isolates related to the ESBL phenotype. This finding is in agreement with another study in Egypt (1). Our results displayed significant relation (*p*<0.05) between *bla*_ CMY-2_ gene with ciprofloxacin and aminoglycoside resistance and *bla*_ CTX-M-14_ gene with only nitrofurantoin resistance. MDR isolates were also related with AmpC beta-lactamase *bla*_ CMY-2_ gene and ESBL production.

To our knowledge, ours is first of its kind investigation which found a relation between ESBL and AmpC β-lactamase with vast number of virulence genes. There was a statistically significant relation between AmpC associated genes such as *bla*_ CMY-2_ with iron acquisition gene *iutA* and *rmpA* involved in hypermucoviscosity, while *bla*_ DHA-1_ had an association with *mrkD* (*p*<0.05). Among ESBL associated genes, *bla*_ CTX-M-14_ showed significant relation with *fimH*. At phenotypic level, ESBL producers allied with adhesin and hypermucoviscosity and AmpC producers were associated with enterobactin and *kpn* (*p*< 0.05). In a study conducted by Ranjbar et al. (27), apart from *rmpA* no significant relations were found between the existence of *bla*_ CTX-M-15_ and virulence genes. Other study furnished the information that the occurrence of β-lactamase or antibiotic resistance genes is related with certain virulence characteristics, particularly *rmpA* and *mrkD* which further validates our findings (28). Nevertheless, previous study did not discover any association between these variables (6).

Our study found twelve combination profiles of the virulence genes. In the current study, we detected the association between AmpC production and one certain virulence profile for the first time which indicates that AmpC-producer isolates carry lipopolysaccharides, enterobactin and, *kpn* as adhesin in the genome (*p*≤0.05). In contrary to a research in Turkey (6) all of our virulence profiles contained at least 4 or more virulence genes.

Distribution and prevalence of virulence genes among clinical samples were compared with a previous study (8). Most of the virulence genes were predominant in isolates obtained from urine samples in our study however, *rmpA* and *iutA* genes related to wound specimen (*p*<0.05). We detected the association between β-lactamase resistance and clinical specimens and indicated that a statistically significant (*p*<0.05) relation exist between *bla*_ CMY-2_ and isolates obtained from wound specimens and *bla*_ DHA-1_ with urine as a source.

Our results indicated that *K. pneumoniae* strains harbored the highest resistance towards cefotaxime, ceftazidime, nitrofurantoin, ciprofloxacin, co-trimaxazole and piperacillin-tazobactam. Ranjbar et al. reported high incidence of antibiotic-resistance in *K. pneumoniae* isolates from Iran and described similar trend in several other countries (27). Infection caused by antibiotic-resistant *K. pneumoniae* has been related to higher fatality rate (27). The present investigation also observed significant relation (*p*<0.05) between the *iutA* (Iron acquisition system) with resistance against 3 ^rd^ generation cephalosporins, aminoglycosides, β-lactams, and fluoroquinolones while, adhesin encoding genes like *mrkD, kpn* and *ycfM* were related with resistance toward one or two antibiotics. Our results are in consistence with a previous study which has presented the correlation between the production of virulence factors and resistance phenotype (27). Khaertynov et al. (28) presented the significant association between *rmpA* and *wcaG* with different antibiotic resistance. Our outcome is in accordance with this study whereby *wcaG* is related to various antibiotic resistance and MDR isolates (*p*<0.05).

## CONCLUSION

The tremendous increase in the incidence of AmpCs and ESBLs were identified concomitantly in our isolates, they may create a real threat for vulnerable patients as majority of ESBL/AmpC-positive isolates were extremely resistant to different antibiotics. The present study could demonstrate that the ESBL/AmpC resistance, virulence profiles and other antibiotic resistance in *K. pneumoniae* not only may affect the patients but also put an alertness for the clinicians to have potential application in designing treatment strategies for controlling its spread within the community.
